# Gastrointestinal endoscopy in Nigeria - a prospective two year audit

**DOI:** 10.11604/pamj.2013.14.22.1865

**Published:** 2013-01-15

**Authors:** Bashiru Omeiza Ismaila, Michael Ayedima Misauno

**Affiliations:** 1 Department of Surgery, Jos University Teaching Hospital

**Keywords:** Gastrointestinal, endoscopic procedures, audit

## Abstract

**Introduction:**

Gastrointestinal (GI) endoscopy is currently performed by different specialties. Information on GI endoscopy resources in Nigeria is limited. Training, cost, availability and maintenance of equipment are some unique challenges. Despite these challenges, the quality and completion rates are important.

**Methods:**

Prospective audit of endoscopic procedures by an endoscopist in a Nigerian hospital over a 24 month period.

**Results:**

One hundred and ninety endoscopic procedures were performed in 187 patients (109 male, 78 female) by a surgeon during this period. Mean age was 47.6 years (range 17 - 90 years). All patients were symptomatic. One hundred and twenty-two procedures (64.2%) were upper GI endoscopy, 52 (27.4%) colonoscopy and 16 (8.4%) sigmoidoscopy. Majority of endoscopies 182 (95.8%) were performed electively and only 7 (3.7%) were therapeutic. Upper GI endoscopy findings included 14 (11.5%) cases of peptic ulcer disease, 5 complicated by gastric outlet obstruction, and 21 (17.3%) cases of upper gastrointestinal cancer. Lower gastrointestinal endoscopy findings included 7 cases of polyps, 3 cases of colorectal cancer and 2 cases of diverticulosis. Commonest lesion on lower GI endoscopy was haemorrhoids (41.7%). Adjusted caecal intubation was 81.4% for colonoscopies performed. Overall adenoma detection rate for male and female patients were 18.2% and 5.3% respectively; in patients over 50 years these were 6.3% and 14.3%. Two complications, rupture of oesophageal varices, and respiratory arrest in bulbar palsy patient occurred.

**Conclusion:**

An endoscopist can perform GI endoscopy effectively in developing countries like Nigeria but attention to equipment need and training is important.

## Introduction

Gastrointestinal endoscopy traditionally performed by gastroenterologists is currently being carried out by different specialists. These include gastroenterologists, surgeons, family physicians and nurses [1–4]. The quality of endoscopic procedure performed is important irrespective of who performs it because of the implications on the diagnosis of gastrointestinal pathology and their treatment. Thus it has become important not only to document the number and spectrum of cases seen but the quality of these examinations. Different standards have already been proposed and adopted in different parts of the world [5–6]. Current interest in quality is more for colonoscopy than for upper gastrointestinal endoscopy [7]. Several publications have evaluated the quality of endoscopy performed by different specialists, but these are in developed countries [3, 8, 9]. In Africa such information is not readily available and even in South Africa one of the more advanced African countries, an audit revealed standards that were below international standards [10]. This audit also revealed delays in provision of endoscopic services, lack of endoscopic equipment, inadequate scope maintenance and disinfection as well as shortage of trained staff. Based on our health indices this is the likely situation across most of sub-Saharan Africa including Nigeria. Can the standards recommended from developed countries be achieved in this setting? This paper is an evaluation of the outcomes of gastrointestinal endoscopies performed by an endoscopist in a public hospital in Nigeria over a 24 month period.

## Methods

This was a prospective quality assurance audit of endoscopic procedures performed by a general surgeon in the Jos University Teaching Hospital. The surgeon received endoscopy training as part of his residency training and also attended some ‘hands on' short courses on endoscopy in the region.

Jos University Teaching Hospital is located in Jos Plateau State in central Nigeria. Gastroenterologists, other surgeons and family physicians also provide endoscopy services in Jos.

Endoscopic procedures were performed by BI in Jos University Teaching Hospital from 11/01/10 to 10/01/12. Routine practice is to perform the procedures on outpatient basis except for emergencies or patients on admission who require endoscopy. Procedures performed in another facility, or performed by another endoscopist were not included.

Data collected prospectively over this period included type of endoscopic procedure performed, indications, patients' symptoms, findings, complications, completion rate, nature of the procedure (elective or emergency) and patients' assessment of the procedure. Although the indication for endoscopy was sometimes more than one, the most important was used. Similarly only the most important finding at endoscopy was used.

Endoscopic findings were determined by the endoscopist and were verified by review of pathology reports where these were available. The adenoma detection rate was determined by dividing the total number of polyps found by total number of colonoscopies performed, stratified according to sex and age.

Upper GI endoscopies were considered complete if the 2nd part of the duodenum was reached in the absence of an obstructing lesion proximally. Ability to visualize, reach and obtain biopsies where there were obstructing lesions was also considered as complete examination. For colonoscopies, complete examination was caecal intubation verified by visualization of the appendiceal orifice and ileocaecal valve, and terminal ileum intubation. Colonoscopies were considered incomplete if the caecum was not reached. Photo documentation was not routinely performed due to absence of the required equipment. Adjusted caecal intubation rate excluded those who had incomplete examination from obstructing lesions, poor bowel preparation and equipment failure.

Procedures were performed initially with Olympus fibre optic endoscopes but later by newer Pentax fibre optic endoscopes when they became available. Before the provision of 2 new endoscopes there were times in the study period when endoscopic procedures could not be performed because of unavailability of functioning endoscopes.

Data were analysed with Epi Info statistical software.

## Results

From January 2010 to January 2012, 192 endoscopic procedures were performed in JUTH by BI. Data for 2 procedures in 2 patients could not be verified leaving 190 procedures carried out on 187 patients. These were 122 (64.2%) upper gastrointestinal endoscopies, 52 (27.4%) colonoscopies and 16 (8.4%) flexible sigmoidoscopies. A total of 57.4% of endoscopies were performed on male patients. The mean age was 47.6 years with a range of 17 to 90 years. Of all the endoscopies 95.8%were performed electively and 96.3% were diagnostic. The characteristics of the endoscopy cases are shown in Table 1.

**Table 1:** Characteristics of cases: N = 190

Characteristic	N (%)
**Sex**	****
Male	109 (57.4)
Female	81 (42.6)
**Presentation**	****
Elective	182 (95.8)
Emergency	8 (4.2)
**Procedure**	****
Upper gastrointestinal	122 (64.2)
Colonoscopy	52 (27.4)
Flexible sigmoidoscopy	16 (8.4)
**Aim**	****
Diagnostic	183 (96.3)
Therapeutic	7 (3.7)

The most common indication for upper GI endoscopy was abdominal pain (54.1%). Other common indications were upper GI bleeding (10.7%), dysphagia (10.7%), reflux related symptoms (9.8%) and recurrent vomiting (9.0%). Other indications are shown in Table 2. The most common indications for lower gastrointestinal endoscopy were rectal bleeding (42.6%) and abdominal pain with or without a mass (22.1%) as shown in Table 3.

**Table 2:** Upper gastrointestinal endoscopy indications and findings: N = 122

Indications	N (%)
Epigastric pain	59 (48.4)
Other abdominal pain	7 (5.7)
Upper gastrointestinal bleeding	13 (10.7)
Recurrent vomiting	11 (9.0)
Dysphagia	13 (10.7)
Reflux related symptoms	12 (9.8)
Abdominal mass	3 (2.5)
Other	4 (3.3)
**Findings**	**N (%)**
Gastritis or duodenitis	42 (34.4)
Normal	19 (15.6)
Oesophageal cancer	13 (10.7)
Gastric/duodenal ulcer	9 (7.4)
Gastric cancer	8 (6.6)
Varices	6 (4.9)
Oesophagitis	6 (4.9)
Benign GOO	5 (4.1)
Benign oesophageal stricture	2 (1.6)
Hiatus hernia	2 (1.6)
Barrett's oesophagus	1
Duodenal obstruction pancreatic cancer	2 (1.6)
Gastric polyp	1 (0.8)
Duodenal polyp	1 (0.8)
GIST	1 (0.8)
Other	4 (3.3)

GIST: gastrointestinal stromal tumour

**Table 3:** Colonoscopy and sigmoidoscopy indications and findings: N = 68

Indications	N (%)
Rectal bleeding	29 (42.6)
Abdominal pain, mass	15 (22.1)
Constipation	8 (11.8)
Anal protrusion	5 (7.4)
Diarrhoea	2 (2.9)
Other	9 (13.2)
**Findings**	****
Normal	26 (38.2)
Haemorrhoids	19 (27.9)
Polyps	7 (10.2)
Fissure, fistula	4 (5.9)
Colorectal cancers	3 (4.4)
Ulcerative colitis	1 (1.5)
Infectious colitis	1 (1.5)
Rectal prolapsed	2 (2.9)
Diverticulosis	2 (2.9)
Other	4 (5.9)

Gastritis or duodenitis was the commonest finding on upper GI endoscopy (34.4%) and 7.4% had peptic ulcer disease (Table 2). Normal findings were the result in 15.6% of the patients. In addition 21 patients had upper gastrointestinal cancer (17.3%).

A total of 68 lower gastrointestinal endoscopies (colonoscopy and flexible sigmoidoscopy) were performed. Although 38.2% of these were normal, haemorrhoids were the most common lesions seen (27.9%). There were 3 (4.4%) cases of colorectal cancers and 7 (10.2%) cases of polyps (excluding those with cancer). One case of ulcerative colitis was seen and 2 patients had diverticular disease (Table 3).

Competency in endoscopy was specifically evaluated for colonoscopy, the more technically demanding procedure. Caecal intubation was successful in 35 (67.3%) cases, when adjusted for poor bowel preparation, colonic strictures and equipment problems; caecal intubation was 81.4% (35/43). A total of 33 colonoscopies were performed on male patients while female patients had 19. Overall adenoma detection rate for male was 18.2% (6/33) while for female it was 5.3% (1/19). The adenoma detection rate for patients older than 50 years was 6.3% (1/16) in males and 14.3% (1/7) in females.

In the 190 endoscopic procedures there were 2 complications, both in upper GI endoscopy. One was variceal rupture during banding with resultant severe haemorrhage and the other was an AIDS patient with bulbar palsy who developed respiratory arrest during endoscopy. Both resulted in mortality. There were no complications associated with the lower GI endoscopic procedures.

## Discussion

This audit shows that endoscopic procedures can be performed with a reasonable degree of competence and safety even in a resource-limited environment like ours. It is, to the best of our knowledge of existing literature, the first attempt by an endoscopist in Nigeria to perform a local quality assurance study.

While there are a number of published literature on the quality of endoscopy in the developed world, [8, 9, 11] the same cannot be said for the developing world [10]. It is difficult to objectively determine good quality endoscopy from a poorly performed one without observing the procedure. Practical measures of quality have been developed by different bodies to assure the quality of an endoscopic procedure. While some of these parameters are in use, there is still need to develop more accurate and suitable measures of quality of endoscopy. The ASGE/ACG Taskforce on Quality in Endoscopy has identified a number of the quality assurance parameters. For upper GI endoscopy for example, the Taskforce stated that apart from carrying out a complete examination with retroflexion in the stomach, one of the basic characteristic of quality upper GI endoscopy is completion of therapeutic procedures like those for upper GI bleeding [12]. For lower gastrointestinal endoscopy, they also stated 90% caecal intubation rate as the standard for colonoscopy and 95% for screening purposes in healthy adult [6]. Criteria set by the US Multi-Society Taskforce on Colorectal Cancer and National Health Service Bowel Cancer Screening Programme are similar [13]. Reports have suggested that some centres achieve this standard for colonoscopy easily [14–16]. However, other more recent reports suggest that this may not widespread [17–19]. While a lot is known about factors which affect the quality of endoscopy in developed countries, for developing countries this is less clear. Major issues are the lack of equipment, manpower, and the high cost of procurement and maintenance of equipment [20, 21].

For upper GI endoscopy which is less technically demanding, complete examination in our study was routine. However therapeutic upper GI procedures, a basic assessment of quality were not routinely performed because of lack of required accessory equipment. Although 3 patients had dilatation of oesophageal obstruction and 2 had banding for oesophageal varices, therapeutic upper GI endoscopy during the study period was not routine. Often patients who would have benefitted from therapeutic endoscopies had other modalities of treatment like surgery. The commonest indication was abdominal pain (mainly epigastric). This is comparable to other studies from the country and region suggesting that abdominal pain is the commonest indication for patients undergoing upper GI endoscopy [22–24]. Gastroduodenitis is also a relatively common finding in this region [22, 23, 25]. In a retrospective study of 989 upper GI endoscopies by surgeons in Jos University Teaching Hospital over an 11 year period, peptic ulcers were seen in 15% of cases [22]. In our series peptic ulcers were seen in 7.4%. This may have been as a result of the more current widespread use of H2 receptor blockers and proton pump inhibitors by patients before endoscopy.

Reports about quality of colonoscopy in Nigeria are not readily available. A retrospective study in Ghana reported complete colonoscopy rate of 30.4% [26]. We achieved an adjusted caecal intubation rate (81.4%) that was lower than the recommended 90% for colonoscopy in the West. We believe that limitation in equipment may be a major factor in our study. While video endoscopes are considered standard equipment in the West and there is a wide range of endoscopes to choose from, we have had only a single functioning fibre-optic colonoscope which in the initial part of the study was one that was taped to stop air leak, reducing flexibility, a practice which would be considered unacceptable but for the constraints in which we work. Another factor may be training; training and retraining have been shown to be useful in improving caecal intubation rates [19]. Factors such as whether the colonoscopy is performed with the intention to treat or per protocol, patients gender, as well as the type of colonoscope used (in women), may also determine caecal intubation rates [27]. Although adenoma detection rates of ≥ 25% and ≥ 15% have been recommended for men and women above the ages of 50 years respectively [13], our detection rates of 6.3% for males and 14.3% for females were lower. Most of the polyps were detected in male patients younger than 50 years. An old study had shown that compared with Negro Americans, Nigerians were likely to be younger and have fewer neoplastic polyps [28]. Although the incidence of colorectal cancer may be rising in Nigeria, there is little doubt that colorectal cancer incidence is lower in West Africa compared to the West [29]. Polyps have been found consistently in the region when colonoscopy is performed [30, 31]. In our study 7 (10.7%) patients who underwent colonoscopy had polyps. Histologies of two polypectomies carried out were juvenile and hyperplastic polyps. We are uncertain whether adenoma detection rate is a useful parameter for assessment of quality of colonoscopy in African patients.

We did not record any complication for colonoscopic procedures which were mainly diagnostic. Generally complication rates for diagnostic colonoscopy are expected to be low [6]. Two patients who had upper GI endoscopy-related deaths are high by any standard. This will have been reduced or eliminated by better patient selection and preparation, availability of resuscitative equipment and better endoscopes.

There is a rising demand for endoscopy in Nigeria, because of its diagnostic and therapeutic applications. The apparent increase in the incidence of colorectal cancer also will require colonoscopy for earlier diagnosis and perhaps prevention [29]. More endoscopists will be required to provide quality endoscopic procedures in the country as current numbers of gastroenterologists are inadequate [21]. To meet this demand, endoscopic procedures will need to be performed by different specialties among which surgeons are an important resource group. A number of the findings require surgical management and this helps to simplify the process of treatment in our environment.

This audit had some limitations. The interruptions of the study as a result of breakdown and unavailability of equipment has contributed to a small sample size during the study period. As this audit process is continuing, it would be observed whether the current trends continue. More local audits of endoscopic procedures across specialties in the region will reveal more accurately the quality of endoscopy.

An issue that needs to be resolved is whether endoscopists in resource poor setting should set local standards for themselves that reflect the realities of the environment in which they practice or adopt standards already recommended for their colleagues in the developed countries. We suggest that rather than set lower standards, we should identify and solve our local problems, so that our standards are at least comparable to that of the developed countries. Local factors that are peculiar and useful for audit in this environment should also be identified.

## Conclusion

This study implies that despite the challenges of poor equipment and training in a resource poor setting, endoscopy can be performed competently by a local endoscopist in Nigeria with good outcomes. However the need for equipment improvement and more training for the provision of quality endoscopic services cannot be overemphasized.
